# Glassy and Polymer Dynamics of Elastomers by ^1^H-Field-Cycling NMR Relaxometry: Effects of Fillers

**DOI:** 10.1021/acs.jpcb.1c00885

**Published:** 2021-04-22

**Authors:** Francesca Nardelli, Francesca Martini, Elisa Carignani, Elena Rossi, Silvia Borsacchi, Mattia Cettolin, Antonio Susanna, Marco Arimondi, Luca Giannini, Marco Geppi, Lucia Calucci

**Affiliations:** † Dipartimento di Chimica e Chimica Industriale, Università di Pisa, via G. Moruzzi 13, 56124 Pisa, Italy; ‡ Istituto di Chimica dei Composti OrganoMetallici, Consiglio Nazionale delle Ricerche, via G. Moruzzi 1, 56124 Pisa, Italy; § Centro per l’Integrazione della Strumentazione Scientifica dell’Università di Pisa (CISUP), Lungarno Pacinotti 43, 56126 Pisa, Italy; ∥ Pirelli Tyre SpA, Viale Sarca 222, 20126 Milano, Italy

## Abstract

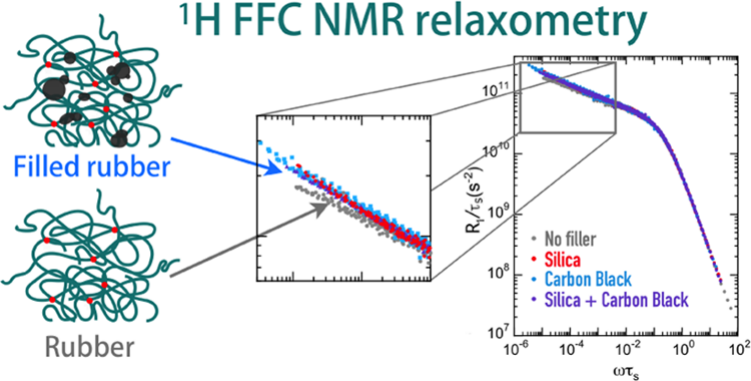

^1^H spin–lattice relaxation rate (*R*_1_) dispersions were acquired by field-cycling (FC) NMR relaxometry between 0.01 and 35 MHz over a wide temperature range on polyisoprene rubber (IR), either unfilled or filled with different amounts of carbon black, silica, or a combination of both, and sulfur cured. By exploiting the frequency–temperature superposition principle and constructing master curves for the total FC NMR susceptibility, χ″(ω) = ω*R*_1_(ω), the correlation times for glassy dynamics, τ_s_, were determined. Moreover, the contribution of polymer dynamics, χ_pol_^″^(ω), to χ″(ω) was singled out by subtracting the contribution of glassy dynamics, χ_glass_^″^(ω), well represented by the Cole–Davidson spectral density. Glassy dynamics resulted moderately modified by the presence of fillers, τ_s_ values determined for the filled rubbers being slightly different from those of the unfilled one. Polymer dynamics was affected by the presence of fillers in the Rouse regime. A change in the frequency dependence of χ_pol_^″^(ω) at low frequencies was observed for all filled rubbers, more pronounced for those reinforced with silica, which suggests that the presence of the filler particles can affect chain conformations, resulting in a different Rouse mode distribution, and/or interchain interactions modulated by translational motions.

## Introduction

Particles of solids, such as carbon black and silica, are usually dispersed in elastomers as reinforcing fillers to improve mechanical, thermal, and rheological properties of the polymeric matrix. The reinforcement process is associated with properties of both the filler particles (morphology, surface area, and composition) and the polymer matrix (microstructure and functional groups). The main molecular mechanisms at the basis of reinforcement of rubbers are the hydrodynamic effect, which results from the introduction of rigid particles in a soft matrix, the elastic properties of the polymer network after vulcanization in the presence of the filler, polymer–filler chemical and physical interactions at the interface, and filler–filler interactions, which give rise to a filler network inside the polymer bulk of increasing importance with increasing the filler content.^1−7^

Elastomer chains can interact with the filler surface and generally exhibit different aggregation states and dynamics compared to the unbound chains in the bulk.^8−13^ The extent of variation of dynamics strongly depends on the distance from the solid surface and temperature, as well as on the characteristics of the polymer and the filler surface. In fact, highly attractive or repulsive filler–polymer interactions result in a slowdown or acceleration of dynamics, respectively. A gradient of dynamics exists on going from the interface to the bulk.^14−20^ The formation of the interfacial layer is also believed to play a crucial role in determining properties of the composite, such as glass-transition temperature, fragility, elastic modulus, and rheological responses, although controversial data were reported in the literature.^7,21−39^ In particular, bridging chains, that is, chains in contact with at least two particles, may interconnect the filler and polymer chains in a network when the distance between adsorption junctions is on the order of that between filler particles or aggregates. Filler–rubber interactions impose constraints to chain dynamics in elastomers,^6,8^ in addition to those arising from entanglements and, in cured compounds, chemical cross-links, which constitute the microscopic origin of macroscopic properties required for specific applications. It is, thereby, of both scientific and technological interest to investigate the effects of fillers on elastomer dynamics.

At a microscopic level, dynamics of polymer melts can be divided into “glassy dynamics” and “polymer dynamics”. Glassy dynamics (also referred to as regime 0) comprises fast motions within the so-called Kuhn segment, characterized by a correlation time τ_s_ and related to the α-relaxation. On the other hand, polymer dynamics concerns collective chain motions occurring over different times and length scales. For polymers with molar mass *M* > *M*_e_ (*M*_e_ is the molar mass between two entanglements), polymer dynamics is most successfully described by the tube reptation (TR) model formulated by Doi and Edwards^40^ on the basis of the De Gennes concept of reptation.^41^ In this model, the complex interactions between a given polymer chain and its neighboring chains, which impose topological constraints on the tagged chain motions, are modeled as a fictitious tube and different regimes are defined for the chain motions far above the glass transition. At short times (ns) and lengths (10^–10^–10^–9^ m), Kuhn segments move freely, subject only to chain connectivity; in this regime (regime I), dynamics is described by the Rouse model.^42^ At longer times (nanoseconds to microseconds) and larger length scales (10^–8^ m), the chain feels that the “tube” and segments undergo “local reptation” or constrained Rouse dynamics (regime II). In regime III, the chain reptates along the tube, until, at very long times (≥ms) and large space scales (≥10^–6^ m), free diffusion is established (regime IV). Characteristic dependences on time of the segmental mean-squared displacement result in the TR model: ⟨*r*^2^(*t*)⟩ ∝ *t*^α^ with α ≤ 0.5 in the subdiffusive regimes, while α = 1 in regime IV. Permanently cross-linked elastomers, such as rubbers obtained by sulfur curing in industrial vulcanization, have usually been considered as entangled polymer melts, although topological constraints due to chemical cross-linking affect the spectrum of polymer dynamics, besides slowing down glassy dynamics.

^1^H-field-cycling nuclear magnetic resonance (FC NMR) relaxometry is a very powerful technique for studying dynamic properties of polymers.^43−49^ This technique measures the dependence of the proton longitudinal relaxation rate (*R*_1_(ω) = 1/*T*_1_(ω)) on the Larmor frequency (*ν* or ω = 2π*ν*), also called nuclear magnetic relaxation dispersion (NMRD)^50^ from 0.01 to 40 MHz using commercial FC NMR relaxometers; this range can be extended at higher frequencies by measuring *R*_1_ with conventional high-field spectrometers, while frequencies down to 100 Hz can be reached with a home-built FC NMR relaxometer compensating the earth’s magnetic field.^51−56^ The frequency–temperature superposition (FTS) principle, usually valid for polymers at temperatures above the glass transition (*T*_g_),^57,58^ can also be adopted to build master curves joining NMRD data acquired at different temperatures;^49,59^ with this assumption, dynamics can be investigated over seven to eight frequency decades. Since ^1^H longitudinal relaxation arises from the modulation of intrachain and interchain dipole–dipole interactions by reorientations and translations of chain segments, NMRD curves reflect the spectrum of motions of ^1^H–^1^H spin pairs in the sample. Indeed, *R*_1_ can be expressed as a linear combination of spectral densities, *J*(ω), the latter being the Fourier transform of the dipolar autocorrelation functions, *C*(*t*). At high frequency and low temperature, where ωτ_s_ ≅ 1, NMRD curves are dominated by fast intrasegment conformational fluctuations connected to glassy dynamics, well described by the Cole–Davidson spectral density.^60^ At lower frequencies and higher temperatures (ωτ_s_ ≪ 1), dipolar translational (*C*_trans_(*t*)) and rotational (*C*_rot_(*t*)) autocorrelation functions associated to polymer dynamics are expressed as proportional to power laws of ⟨*r*^2^(*t*)⟩. In particular, in the Rouse regime, where ⟨*r*^2^⟩ ∝ *t*^1/2^, *C*_rot_(*t*) ∝ *t*^–1^ and *C*_trans_(*t*) ∝ *t*^–3/4^, thus resulting in the dependence of the corresponding spectral densities on ln(ω) and on ω^–1/4^.^47,48^ Thereby, different dependences of *R*_1_ on the frequency are found in different regimes, depending on the relative weight of the intra- and interchain contributions.^46−48,61^ Since glassy dynamics is strongly prevalent over polymer dynamics,^44^ the latter can only be correctly investigated after separation of these two components in FC NMR data, as pointed out by Rössler and co-workers.^45,47,62−68^

^1^H FC NMR relaxometry was successfully applied to investigate glassy and polymer dynamics in polymer melts^44−48,62−67^ and, less extensively, in cross-linked elastomers.^69−73^ In particular, in previous work, we investigated the effect of introducing cross-links by sulfur curing on glassy and polymer dynamics of elastomers.^73^ A progressive slowing down of glassy dynamics was found on increasing cross-linking density for polyisoprene, polybutadiene, and poly(styrene-*co*-butadiene) rubbers, paralleled by an increase of *T*_g_. Power law dependences ascribable to polymer dynamics in regime I and II of the TR model were observed in the investigated temperature and frequency range for all of the elastomers before curing, whereas only regime I was found for vulcanized rubbers, indicating that permanent cross-linking results either in suppression or in slowing down of entangled dynamics, which therefore occurs at frequencies below those accessed in our study.

In the present work, ^1^H FC NMR relaxometry is applied to cross-linked polyisoprene rubber (IR) obtained by sulfur curing, either unfilled or filled with carbon black, silica, or a combination of them, to investigate the effects of filler particles on glassy and polymer dynamics. Carbon black is the most ancient and widely employed filler in rubber technology.^74^ Its reinforcing effect is due to the fractal nature of particle aggregates and filler networks present in the rubber bulk,^75−77^ as well as due to polymer–filler interactions.^78,79^ Silica has been later introduced in rubber technology to achieve specific performances such as low hysteresis.^80−82^ However, the silica surface is characterized by the presence of silanol groups, which reduce the affinity toward nonpolar hydrocarbon elastomers with respect to carbon black and tends to form particle aggregates, with a consequent reduction of the reinforcement effect. To avoid these drawbacks, the silica surface is often modified by functionalization of silanol groups with silanes. Mixed reinforcing fillers have also been proposed to reduce filler–filler aggregation and obtain a synergetic response.^83^ Here, ^1^H FC NMR relaxometry measurements were performed between 0.01 and 35 MHz over a broad temperature range. NMRD curves were compared with those previously reported for natural rubber filled with carbon black.^70,71^ The application of a procedure including the construction of NMR susceptibility master curves and the disentanglement of contributions to longitudinal relaxation from glassy and polymer dynamics, here adopted for the first time for filled rubbers, allowed a detailed investigation of the effects of filler particles on glassy dynamics and polymer dynamics in the Rouse regime.

## Experimental Section

### Samples

All samples (Table 1) were provided by Pirelli Tyre SpA (Milano, Italy). High-molar-mass *cis*-1,4-polyisoprene (≥96% cis, *M*_w_ = 1.49 × 10^6^ g/mol, *M*_n_ = 8.41 × 10^5^ g/mol, *T*_g_=208 K) was used as basis for the cross-linked rubbers. The cross-linking reaction was performed using a standard system of *N*-*tert*-butyl-2-benzothiazolesulfenamide (TBBS, 3 phr) and sulfur (S, 2 phr), activated by zinc oxide (3 phr) and stearic acid (2 phr). Finally, *N*-(1,3-dimethylbutyl)-*N*′-phenyl-*p*-phenylenediamine (6PPD) was used as an antioxidant (2 phr). The curing package was kept constant for all compounds. Carbon black N330 (surface area: 77 m^2^/g) was used for samples IR_S2_CBX, where X is the carbon black (CB) content in phr, and for IR_S2_Si15_CB20. Commercial silica (Ultrasil 7000; surface area: 175 m^2^/g; silanol content: 2.7 mmol/g) and bis(triethoxysilylpropyl)tetrasulfide (TESPT) were used to prepare IR_S2_Si15_CB20 and IR_S2_Si50. All compounds were mixed in a 1.5 L internal mixer (Harburg-Freudenberger, Hamburg, Germany) in a two-step mixing process. In the first step, all ingredients except the vulcanization system were mixed for 200 s reaching a dumping temperature of approximately 140 °C with a rotor speed of 75 rpm; silica compounds were mixed for only 120 s. In the second step, the vulcanization system was added and the compound was finalized by mixing for 120 s at 40 °C with a rotor speed of 50 rpm, and the maximum dumping temperature was set at 110 °C. All polymers were vulcanized at *T*_vulc_= 150 °C and at the optimum cure time, i.e., the time corresponding to the maximum torque.

**Table 1:** Composition and Vulcanization Conditions of the Investigated Samples and ^1^H Residual Dipolar Couplings (*D*_res_) Determined by MQ-NMR Experiments

sample	sulfur (phr)^a^	*T*_vulc_ (°C)	carbon black (phr)	silica (phr)	TESPT (phr)	*D*_res_/2π (Hz)^b^
IR_S2	2	150				211
IR_S2_CB20	2	150	20			235
IR_S2_CB40	2	150	40			228
IR_S2_CB60	2	150	60			232
IR_S2_CB80	2	150	80			227
IR_S2_Si50	2	150		50	4	210
IR_S2_Si15_CB20	2	150	20	15	1.2	235

a phr = parts per hundred rubber.

b Errors are of ±10 Hz.

Glass-transition temperatures (*T*_g_) were determined by differential scanning calorimetry (DSC) using a DSC Mettler-Toledo 822e instrument. Thermal cycles between 183 and 323 K were performed and the cooling/heating rate was 10 K/min. *T*_g_ was determined as the intersection point of the two tangents to the DSC curve at the endothermic step. For all samples, *T*_g_ was 216 ±1 K.

### ^1^H MQ-NMR Measurements

^1^H Multiple Quantum (MQ) NMR experiments were carried out on a spectrometer made of a Stelar PC-NMR acquisition system and a Niumag permanent magnet, working at a ^1^H Larmor frequency of 20.8 MHz, equipped with a 5 mm static probe head and a Stelar variable temperature controller. A time incremented 1 cycle version of the improved MQ Baum-Pines pulse scheme was used.^84^ All measurements were performed at room temperature, using a 90° pulse of 3 μs and a recycle delay of 0.5 s. For all experiments, 64 scans were accumulated. The double quantum build-up curves were obtained by measuring the free induction decay signal intensity at increasing values of the cycle time. Residual dipolar coupling (*D*_res_) values were obtained by fitting the build-up data using the second-moment approximation corrected by a decaying Weibullian function. *D*_res_ values are reported in Table 1.

### ^1^H FC NMR Measurements

^1^H *R*_1_ values were measured at different temperatures in the 0.01–35 MHz Larmor frequency range using a Spin Master FFC-2000 FC NMR relaxometer (Stelar SRL, Mede, Italy). The prepolarized and nonprepolarized pulse sequences were used below and above 12 MHz, respectively.^50^ The polarizing and detection frequencies were 25.0 and 16.3 MHz, respectively. The switching time was 3 ms and the 90° pulse duration 9.8 μs. A single scan was acquired. All of the other experimental parameters were optimized for each measurement. All of the ^1^H magnetization curves *vs* time were monoexponential within experimental error and the errors on *R*_1_ were always lower than 3%. For measurements, samples were cut into small pieces and introduced in a 10 mm diameter glass tube. The temperature was controlled within ±0.1 °C with a Stelar VTC90 variable temperature controller.

## Results and Discussion

**Figure 1:**
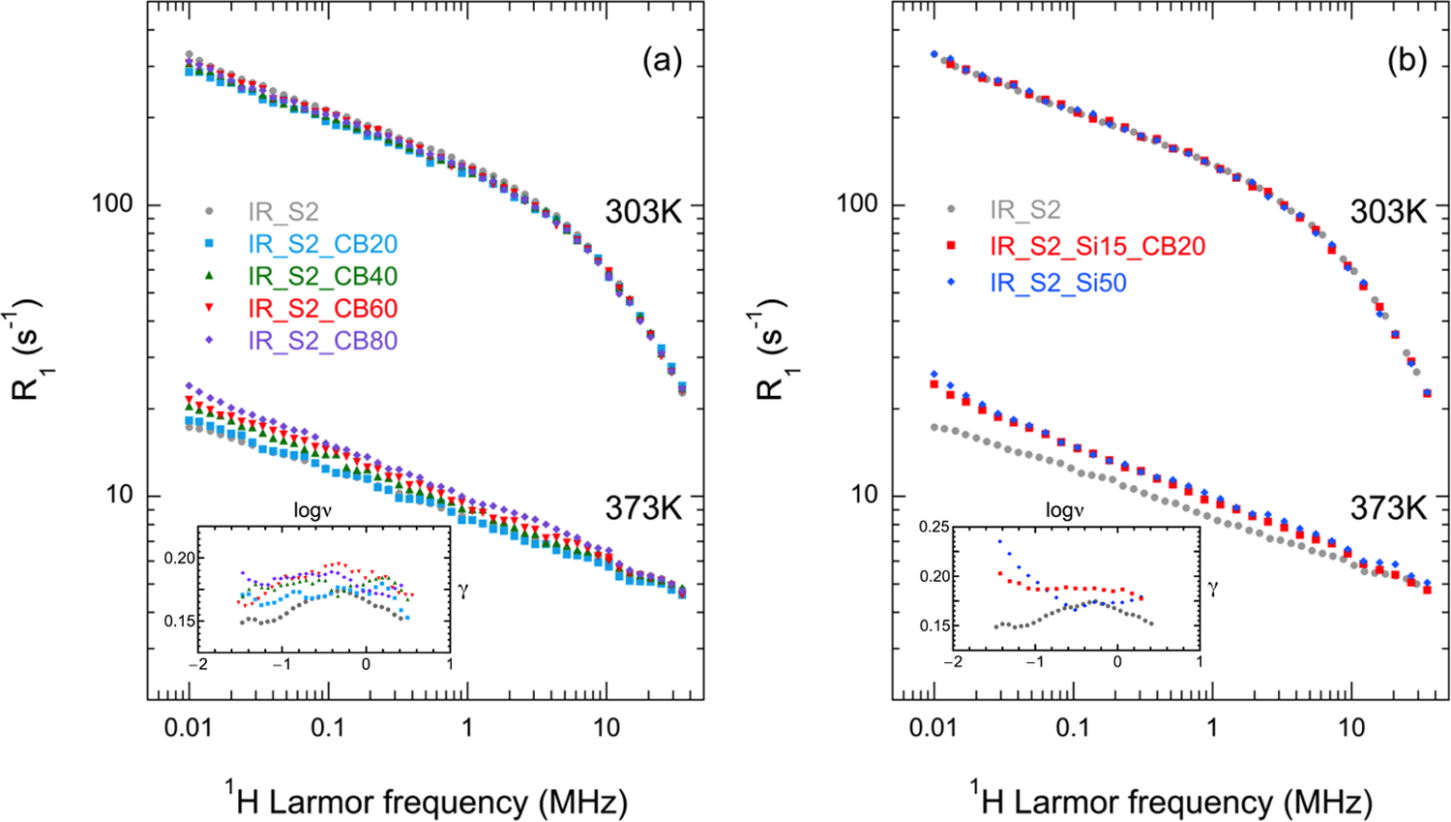
^1^H NMRD curves of unfilled and filled IR rubbers recorded at 303 and 373 K. In the insets, values of the γ exponent determined by applying the derivative method reported in ref (68) to the NMRD curves at 373 K are shown.

^1^H FC NMR relaxometry experiments were performed at 303 and 373 K on IR rubbers filled with different amounts of carbon black (IR_S2_CB20, IR_S2_CB40, IR_S2_CB60, and IR_S2_CB80), silica (IR_S2_Si50), or a mixture of silica and carbon black (IR_S2_Si15_CB20); NMRD curves are reported in Figure 1 together with those of unfilled IR rubber vulcanized in the same conditions (IR_S2). At 303 K, all samples show NMRD curves with a power law dependence on frequency (*R*_1_(ω) ∝ ω^–γ^) at lower frequencies (γ = 0.16–0.18) and a dispersion at higher frequencies. The observed exponents are similar to those reported in the literature for high-molar-mass (*M* > *M*_e_) polyisoprene^64−66,68−70,85^ and IR rubbers,^73^ and can be ascribed to the Rouse regime (regime I of the TR model), while the dispersion corresponds to glassy dynamics (regime 0). Small differences were found between the NMRD curves of filled rubbers and that of IR_S2.

At 373 K, a power law dependence of *R*_1_ on the Larmor frequency is observed for all samples, although with a larger γ exponent for filled rubbers, as shown in the insets of Figure 1. Indeed, γ ranges from 0.15 to 0.17 for IR_S2 and shows values up to 0.19 for rubbers filled with carbon black and up to 0.23 for the rubber reinforced with silica. Moreover, when compared to IR_S2, filled rubbers show higher *R*_1_ values at all frequencies, progressively increasing with the filler content in the case of carbon black. The bigger differences observed between samples at 373 K with respect to 303 K can be ascribed to the fact that at a higher temperature the mobility of chains is increased and motions occur on larger time and length scales; thereby, it is expected that they experience the constraints introduced by filler particles more strongly. An increase of *R*_1_ at high temperature and low frequencies with increasing the filler content was also reported by Kariyo and Stapf for natural rubber filled with carbon black;^70,71^ however, no clear effects on dispersion slopes were reported in this case because of the smaller filler content (up to 50 phr) and lower temperatures (up to 333 K) investigated.

**Figure 2:**
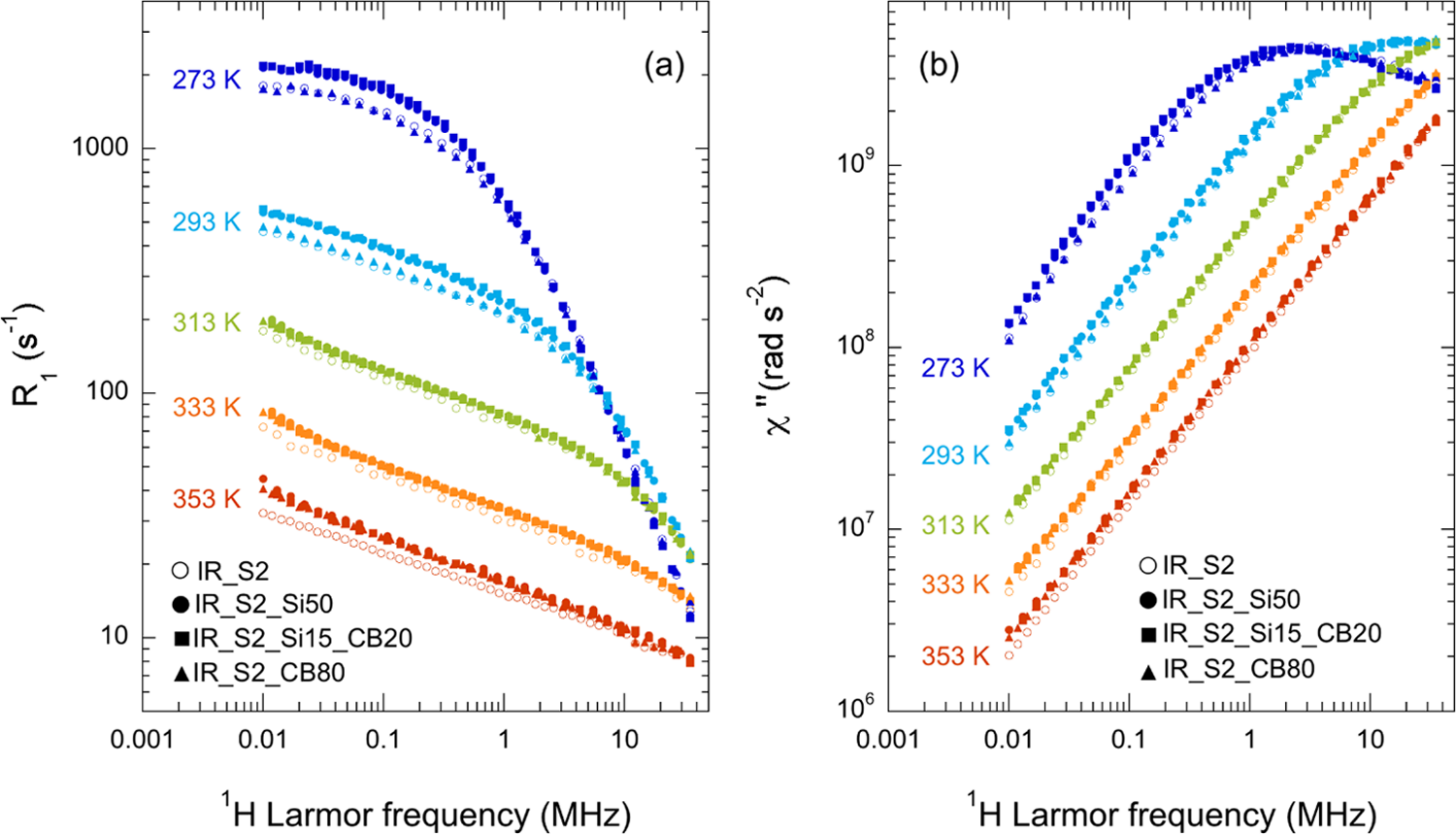
^1^H (a) NMRD and (b) susceptibility curves of IR_S2 (empty circles), IR_S2_Si50 (full circles), IR_S2_Si15_CB20 (full squares), and IR_S2_CB80 (full triangles) at the indicated temperatures.

To better investigate the effect of fillers, ^1^H FC NMR relaxometry experiments were performed at different temperatures between 263 and 373 K on IR_S2 and on the filled rubbers for which stronger effects of fillers on NMRD curves were observed at 303 and 373 K, i.e., IR_S2_CB80, IR_S2_Si50, and IR_Si15_CB20. A selection of NMRD curves is shown in Figure 2a. At low temperatures (*T* < 293 K), NMRD curves are dominated by glassy dynamics. By increasing the temperature, power law dependencies are observed at lower frequencies due to polymer dynamics. For *T* ≥ 353 K, only the power law dependence ascribed to Rouse dynamics is observed, with filled rubbers showing larger γ values with respect to the unfilled one. For all samples, crossover points between regimes shift to higher frequencies by increasing the temperature.

Small but significant differences are observed among the NMRD curves of the different samples as a function of temperature. In particular, with respect to IR_S2, IR_S2_CB80 shows slightly lower *R*_1_ values for *T* ≤ 283 K, but slightly higher for *T* ≥ 293 K, ascribable to faster and slower dynamics, respectively. Rubbers filled with silica (IR_S2_Si50) or silica and carbon black (IR_S2_Si15_CB20) show higher *R*_1_ values with respect to IR_S2 at all temperatures and frequencies, ascribable to slower dynamics of polymer chains.

To better investigate glassy dynamics, we passed to the susceptibility representation by defining a (non-normalized) FC NMR susceptibility χ″(ω) = ω*R*_1_(ω).^47,49,59^ Susceptibility curves exhibit maxima when the condition ωτ_s_ ≅ 1 is matched, and power law dependences of the type χ″(ω) ∝ ω^1−γ^ for ωτ_s_ < 1. As shown in Figure 2b, χ″ maxima were observed at low temperature for all samples, the maximum position being shifted to lower frequencies for IR_S2_Si50 and IR_S2_Si15_CB20 and at a slightly higher frequency for IR_S2_CB80 with respect to IR_S2.

**Figure 3:**
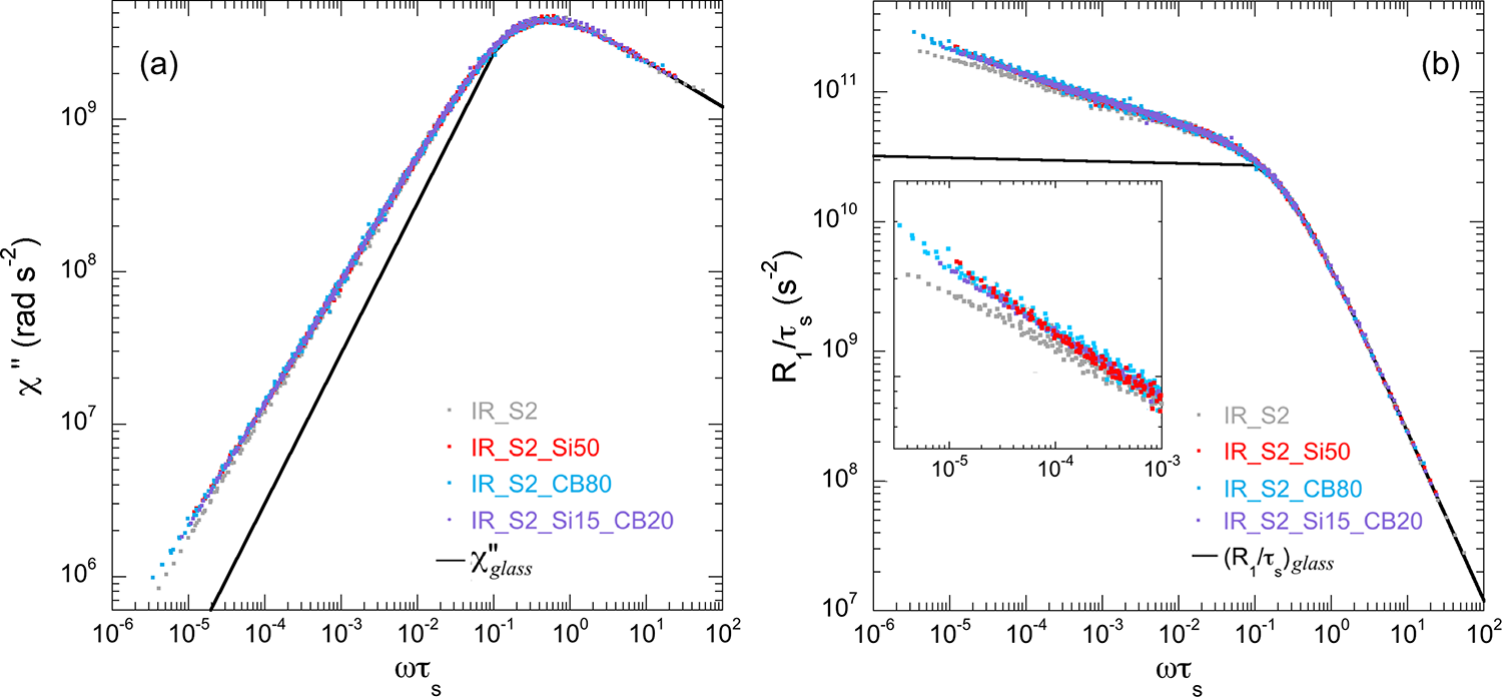
(a) χ″ and (b) *R*_1_/τ_s_ master curves of IR_S2_Si50, IR_S2_CB80, and IR_S2_Si15_CB20 compared to that of IR_S2. Black lines represent NMR susceptibility and *R*_1_/τ_s_ components arising from glassy dynamics. An expansion of *R*_1_/τ_s_ at low reduced frequencies is shown in the inset of panel (b).

**Figure 4:**
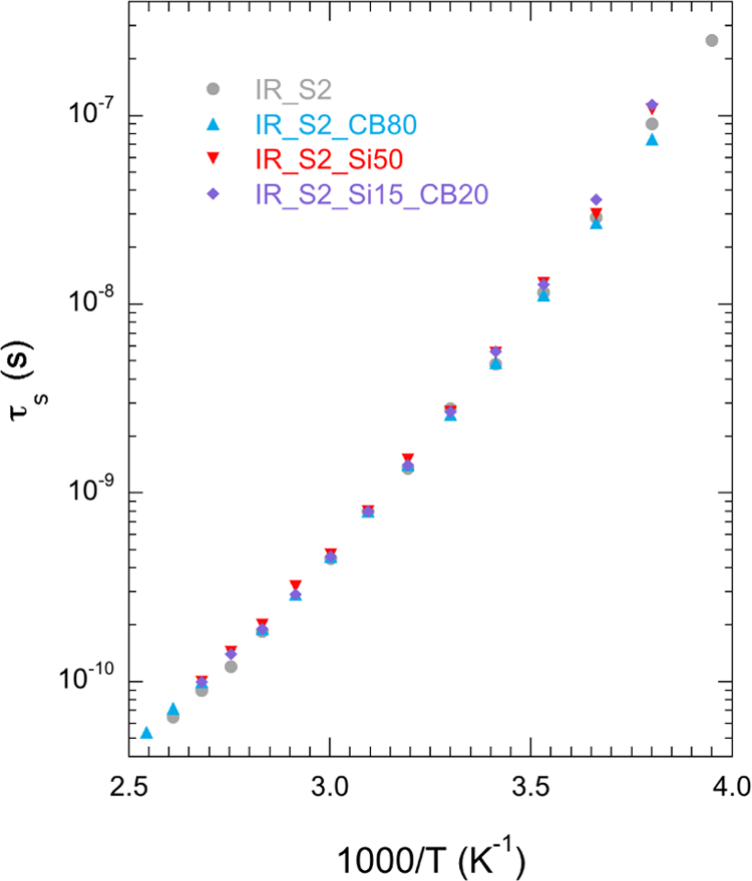
Correlation times for glassy dynamics (τ_s_) *vs* inverse temperature for IR_S2, IR_S2_CB80, IR_S2_Si50, and IR_S2_Si15_CB20.

Quantitative information on glassy dynamics could be obtained by building χ″(ωτ_s_) master curves as a function of frequency reduced by the correlation time of glassy dynamics, τ_s_, on the basis of the FTS principle. At low temperatures (*T* ≤ 273 K), the maxima and the high-frequency branches of the χ″(ω) curves are observed for all samples (Figure 2b), which are essentially determined by glassy dynamics (χ_glass_^″^(ω)). Therefore, τ_s_ can be determined by fitting the high-frequency branch (ωτ_s_ ≥ 1) of the NMR susceptibility curves to the equation
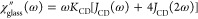
1where *K*_CD_ is a proportionality constant that depends on second moment of relevant dipolar interactions and *J*_CD_(ω) is the Cole–Davidson (CD) spectral density function^60^
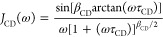
2This function is generally used to describe glassy dynamics of polymers; it is characterized by the parameter β_CD_ (0 < β_CD_ ≤ 1) and by the correlation time τ_CD_, which are connected to τ_s_ by the relation τ_s_ = β_CD_τ_CD_. Good fittings were obtained in all cases with β_CD_ = 0.3 and best fit τ_CD_ values were used to determine τ_s_. For *T* ≥ 283 K, τ_s_ values were determined as the shift factors of the frequency axis used to make χ″(ω) curves to overlap those at lower temperatures, taken as reference. The obtained χ″(ωτ_s_) master curves, covering a frequency range of six to seven decades, are reported in Figure 3a for all samples, while values of τ_s_ are shown in Figure 4.

As expected on the basis of the higher γ values determined for the NMRD curves at high temperature, χ″(ωτ_s_) master curves of filled rubbers show a deviation from that of unfilled IR_S2 at the lowest frequencies. This deviation, which can be better highlighted using the “spectral density” master curve (Figure 3b) obtained by dividing χ″(ωτ_s_) by ωτ_s_, is a clear indication that fillers change the spectrum of chain motions in the Rouse regime.

The correlation times for glassy dynamics (Figure 4) are quite similar for all samples, although systematically longer for rubbers containing silica (IR_S2_Si50 and IR_S2_Si15_CB20) with respect to the unfilled rubber. On the other hand, IR_S2_CB80 shows τ_s_ values slightly longer and shorter than those of IR_S2 at temperatures higher and lower than 283 K. The small differences observed between τ_s_ values are in agreement with the *T*_g_ values determined by DSC for the different samples, which are equal within the experimental error (216 ± 1 K). Similarly, small variations of the correlation time for glassy dynamics, and correspondingly of *T*_g_, were observed by other authors using different experimental techniques.^22,28−30,32,36^ It must be pointed out that all of the investigated samples show very similar values of *D*_res_, measured by MQ-NMR experiments^86^ (Table 1), in agreement with results reported by other authors.^87^*D*_res_ gives a measure of local chain order arising from anisotropic fast segmental motions of the elastomer chains, which, in turn, is due to constraints to the chain motions. In reinforced cross-linked elastomers, *D*_res_ results from constraints imposed by polymer-chain entanglements, chemical cross-links formed by vulcanization, and adsorption or interactions between elastomer and filler particles. The insignificant differences of *D*_res_ found among the samples indicate that the overall cross-link density resulting from all of these constraints does not change much, in agreement with the negligible differences observed for glassy dynamics.

**Figure 5:**
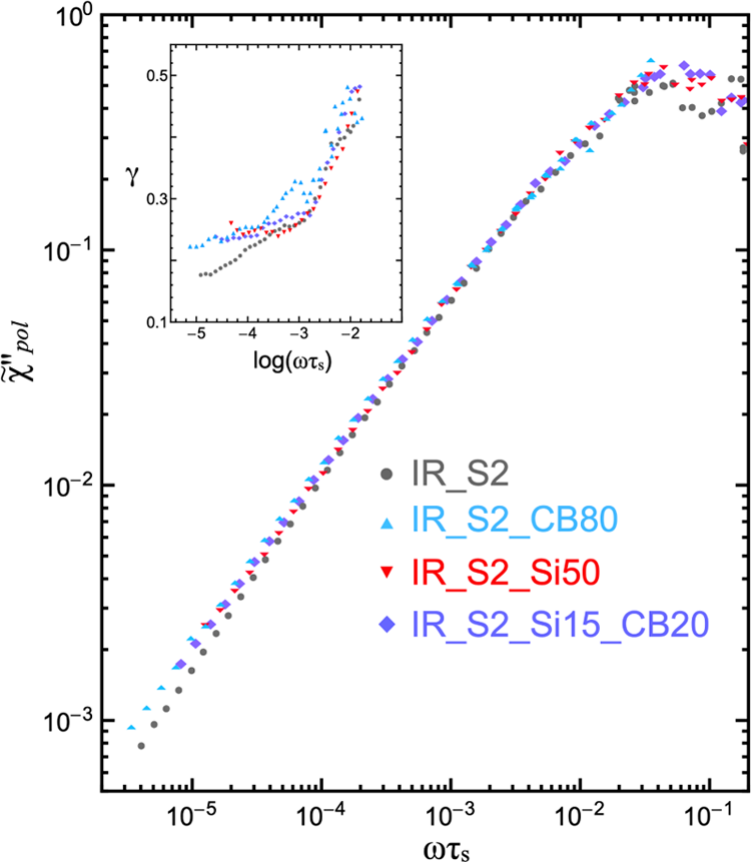
Normalized polymer spectra χ̃″_pol_(ωτ_s_) of IR_S2, IR_S2_CB80, IR_S2_Si50, and IR_S2_Si15_CB20, obtained from the susceptibility master curves after eliminating redundant data. Each spectrum was normalized to provide an integral equal to π/2. Values of the γ exponent, determined by applying the derivative method reported in ref (68), are shown in the inset.

To better investigate the effect of fillers on polymer dynamics and obtain power law exponents not affected by glassy dynamics, “polymer spectra” were singled out from the susceptibility master curves shown in Figure 3a following a spectral decomposition procedure similar to that introduced by Rössler and co-authors^62^ and already adopted by us for cross-linked elastomers.^73^ In this procedure, the polymer spectrum, χ_pol_^″^(ωτ_s_), is obtained by subtracting the contribution of glassy dynamics, χ_glass_^″^(ωτ_s_), from each χ″(ωτ_s_) master curve, as described by eq 1 with the Cole–Davidson spectral density (eq 2). The assumption of statistical independence and time-scale separation between glassy and polymer dynamics was made so that χ″(ωτ_s_) can be written as the sum of glassy and polymer contributions
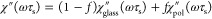
3where *f* represents the fractional contribution of polymer dynamics. Moreover, it was assumed that the high-frequency branch of χ″(ωτ_s_) (ωτ_s_≥1) is practically coincident with χ_glass_^″^(ωτ_s_), the contribution of polymer dynamics at high frequencies being negligible. The polymer spectra normalized to provide an integral equal to π/2 (χ̃″_pol_(ωτ_s_)) are shown in Figure 5 for all samples. As previously observed for high-molar-mass polyisoprene melts^65^ and cross-linked IR,^73^ the values of γ determined from the polymer spectra are different from those found from the NMRD and susceptibility curves, indicating a significant contribution of glassy dynamics to ^1^H longitudinal relaxation at low frequencies. Moreover, different power law dependences of χ̃″_pol_(ωτ_s_) on reduced frequency are found for filled IR samples with respect to the unfilled one in the Rouse regime. In particular, as shown in the inset of Figure 5, at low reduced frequencies (ωτ_s_ ≤ 0.001), γ ranges between 0.22 and 0.26 for IR_S2_CB80, IR_S2_Si50, and IR_S2_Si15_CB20 while it goes down from 0.24 to 0.18 on decreasing the frequency for IR_S2. These changes of γ, resulting from the addition of fillers to rubber, are analogous to those observed for stretched rubbers,^69−71,88^ polymer films on surfaces,^89,90^ or for a soft polymer confined between lamellae of a rigid polymer in diblock and triblock copolymers.^71,91^ In all cases, geometrical confinement is imposed on chain motions either by external forces or by interactions with solid surfaces. Therefore, the different values of γ could be associated with changes in chain conformations induced by interactions with surfaces,^92,93^ resulting in partial chain alignment close to the surface, and in different mode distributions at lengths on the order of the length scale of Rouse motions. It must be pointed out that an increase of γ in the Rouse regime was found by molecular dynamics calculations on decreasing chain flexibility in entangled polymer melts.^94^ Moreover, we cannot exclude that dipole–dipole interchain interactions, favored in the presence of filler particles, also contribute to the increase of γ.^95^ On the other hand, such subtle effects on chain properties are not detected by *D*_res_ measurements (see above).

Going back to NMRD curves at 373 K for rubbers filled with increasing amounts of carbon black, the progressive increase of γ (see the inset of Figure 1a) can be ascribed to the increase of interacting chains with respect to bulk chains. Silica nanoparticles and, most interesting, mixed carbon black and silica seem to give more effective interactions (see the inset of Figure 1b), most probably because of the larger interfacial area of the filler particles.

## Conclusions

Glassy and polymer dynamics were investigated on filled IR rubbers of technological interest for the tire industry. ^1^H FC NMR relaxometry measurements over a wide range of temperatures and frequencies, combined on the basis of the FTS principle, allowed dynamics to be carefully investigated over a quite broad time scale, ranging from local segmental motions within the Kuhn segment to Rouse motions under the constraints imposed by entanglements, sulfidic cross-links formed during vulcanization, and fillers. A moderate effect of fillers on glassy dynamics was found, indicating that intrasegmental motions are only slightly perturbed by the presence of fillers.

The effect of fillers on collective chain dynamics was investigated in detail by separating the contributions of polymer and glassy dynamics to ^1^H longitudinal relaxation, using a procedure adopted for the first time in this work for filled elastomers. In the investigated frequency range, polymer dynamics in the Rouse regime was observed for all rubbers, with small but appreciable changes induced by the presence of fillers in the spectrum of Rouse modes, analogous to that found for stretched rubbers, polymer films on surfaces, or stiffened chains. A stronger effect was observed for silica and mixed silica/carbon black with respect to carbon black.

^1^H FC NMR relaxometry revealed a powerful technique to investigate the effects of fillers on polymer dynamics in the time and length scales of Rouse modes, a dynamic range not easily accessed by other techniques.^96^ Measurements at lower frequencies with a home-built FC NMR relaxometer would give information on longer time and length scales, thus helping in further understanding the effect of fillers on polymer dynamics.

## Abbreviations

FC: field cycling
NMRD: nuclear magnetic relaxation dispersion
IR: isoprene rubber
